# ﻿The mid-Cretaceous crown wasp genus †*Tumidistephanus* Ge & Tan: discovery of the first male and a new species (Hymenoptera, Stephanidae)

**DOI:** 10.3897/zookeys.1248.157257

**Published:** 2025-08-04

**Authors:** Si-Xun Ge, Li-Li Ren, Jiang-Li Tan

**Affiliations:** 1 College of Forestry, Beijing Forestry University, Beijing 100083, China Beijing Forestry University Beijing China; 2 Shaanxi Key Laboratory for Animal Conservation / Key Laboratory of Resource Biology and Biotechnology in Western China, College of Life Sciences, Northwest University, 229 North Taibai Road, Xi’an, Shaanxi 710069, China Northwest University Xi’an China

**Keywords:** Apocrita, fossil, †Lagenostephaninae, mid-Cretaceous Burmese amber, parasitoid wasps, sexual dimorphism

## Abstract

The fossil crown wasp †*Tumidistephanusepimetheus***sp. nov.** is described based on a well-preserved male specimen embedded in mid-Cretaceous Burmese amber. This discovery adds to the growing diversity of the extinct subfamily †Lagenostephaninae, representing its fourth known species. Notably, it constitutes only the second male specimen described within this lineage, offering valuable insight into the morphological characteristics and potential sexual dimorphism in early crown wasps.

## ﻿Introduction

The family Stephanidae (Hymenoptera: Apocrita), representing an early-diverging lineage of parasitoid wasps (Sharkey et al. 2012; Branstetter et al. 2017), is characterized by the tuberculate head capsule, an elongated pronotum, and a dentate hind femur (van Achterberg 2002; Engel and Grimaldi 2004; Binoy et al. 2020a, b; Ge et al. 2021a, b, 2022). Although extant species are cosmopolitan in distribution, primarily in tropical and subtropical regions, they are infrequently collected, a rarity that may reflect not only their actual scarcity but also limitations in targeted sampling methods (Taylor 1967; Kirk 1975). Given their basal phylogenetic position and evolutionary significance, the limited fossil record of this family is particularly noteworthy. Most fossil representatives have been recovered from mid-Cretaceous Burmese and Eocene Baltic amber deposits (Engel and Grimaldi 2004; Li et al. 2017; Ge et al. 2023), providing rare but important insights into the historical diversity of the group.

Recent research has significantly enhanced our understanding of the diversity of Stephanidae, particularly through the discovery and description of fossil taxa. The extinct subfamily †Lagenostephaninae was recently erected to accommodate several Cretaceous genera, including †*Lagenostephanus* Li, Rasnitsyn, Shih & Ren, †*Tumidistephanus* Ge & Tan, and †*Neurastephanus* Ge & Tan (Ge et al. 2023). Among them, †*Tumidistephanus*, is notable for its conspicuously robust hind femur and tibia, along with a distinctive wing venation. The type species, †*T.prometheus* Ge & Tan, described from mid-Cretaceous Burmese amber, has offered valuable insights into the early diversification of the family (Ge et al. 2023).

Here, we describe a new species of †*Tumidistephanus*, *T.epimetheus* Ge & Tan, sp. nov., based on a well-preserved male specimen embedded in mid-Cretaceous Burmese amber. This represents the second known male of the subfamily †Lagenostephaninae and contributes important morphological data for interpreting early sexual dimorphism and character evolution within crown wasps.

## ﻿Materials and methods

The amber deposit containing †*Tumidistephanusepimetheus* sp. nov. was discovered in the Hukawng Valley, Tanai township, Myitkyina district of Kachin state, Myanmar. The amber was mined prior to 2017, and the type specimen described in this study has been deposited at the College of Forestry, Beijing Forestry University, China (BFU). The specimen was examined and photographed using a Canon G9 camera attached to an Olympus CX31 microscope, with final images enhanced for clarity and brightness in Adobe Photoshop CC. Morphological terminology follows Ge et al. (2023).

## ﻿Taxonomy

### ﻿Order Hymenoptera Linnaeus, 1758

**Superfamily Stephanoidea Leach, 1815**

**Family Stephanidae Leach, 1815**

**Subfamily †Lagenostephaninae Ge & Tan, 2023**

#### ﻿Genus † Tumidistephanus

Taxon classificationAnimaliaHymenopteraStephanidae

Ge & Tan, 2023

D1AEC822-31F2-5EDE-B7AE-1BEFF8C4EA38

†Tumidistephanus Ge & Tan, 2023: 819–844 (original description, keyed).

##### Type species.

†*Tumidistephanusprometheus* Ge & Tan, 2023.

##### Diagnosis.

Mesosoma robust. Metafemur distinctly swollen medially, appearing nearly oval in shape. Metafemur ventrally with two prominent and at least five medium-sized teeth. Female metatarsus five segmented. Forewing in females with veins Rs + M and 1Cu non-parallel in females; junction between 2-Rs, M, and 2Rs+M distinctly disconnected, forming a conspicuous gap. First metasomal tergite (T1) and sternite (S1) not fused laterally. Ovipositor length nearly as long as metasoma.

#### ﻿† Tumidistephanus epimetheus

Taxon classificationAnimaliaHymenopteraStephanidae

Ge & Tan sp. nov.

182FC848-1FE8-5983-9102-5F56C406F561

https://zoobank.org/C47E9CF8-E076-4707-8D4A-30977E5ABE67

Figs 1 , 2 , 3

##### Material examined.

***Holotype*** • ♂, Hukawng Valley, Tanai township, Myitkyina district of Kachin state, Myanmar) (BFU).

##### Etymology.

The species name is derived from Epimetheus, the younger brother of Prometheus in ancient Greek mythology.

##### Diagnosis.

Head subcircular, with seven distinct coronal teeth; forewing with 1-M approximately 0.5 × as long as 1-Rs and slightly curved; veins Rs + M and 1Cu parallel; vein 2Rs+M nearly absent, with veins 2-Rs and the apical abscissa of M distinctly unconnected. Metafemur markedly robust, with two prominent and five medium-sized teeth.

**Figure 1.**
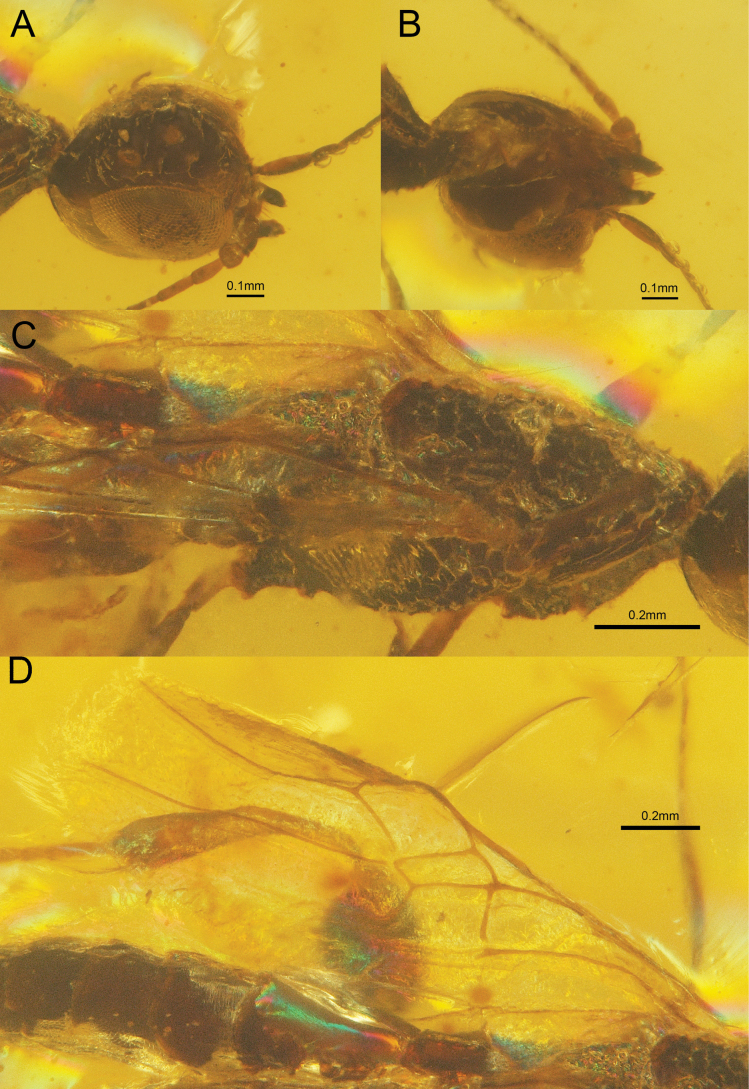
†*Tumidistephanusepimetheus* Ge & Tan, sp. nov. holotype ♂. A. Head, dorsal-lateral view; B. Head, ventral view; C. Mesosoma, dorsal-lateral view; D. Wings.

##### Description.

***Holotype*.** • ♂, length of body about 3.25 mm (excluding antennae). Forewing length about 1.78 mm.

***Head*.** Antenna filiform, elongate, composed of at least 19 flagellomeres; first flagellomere elongated and robust, second flagellomere comparatively short. Head broadly subcircular; frons coarsely obliquely to transversely rugose near anterior coronal tooth; vertex with seven distinct tubercles, transversely carinate posterior to the anterior coronal teeth, carinae not extending to the occipital carina. Occipital carina obsolete; temple moderately expanded posterior to compound eye.

**Figure 2.**
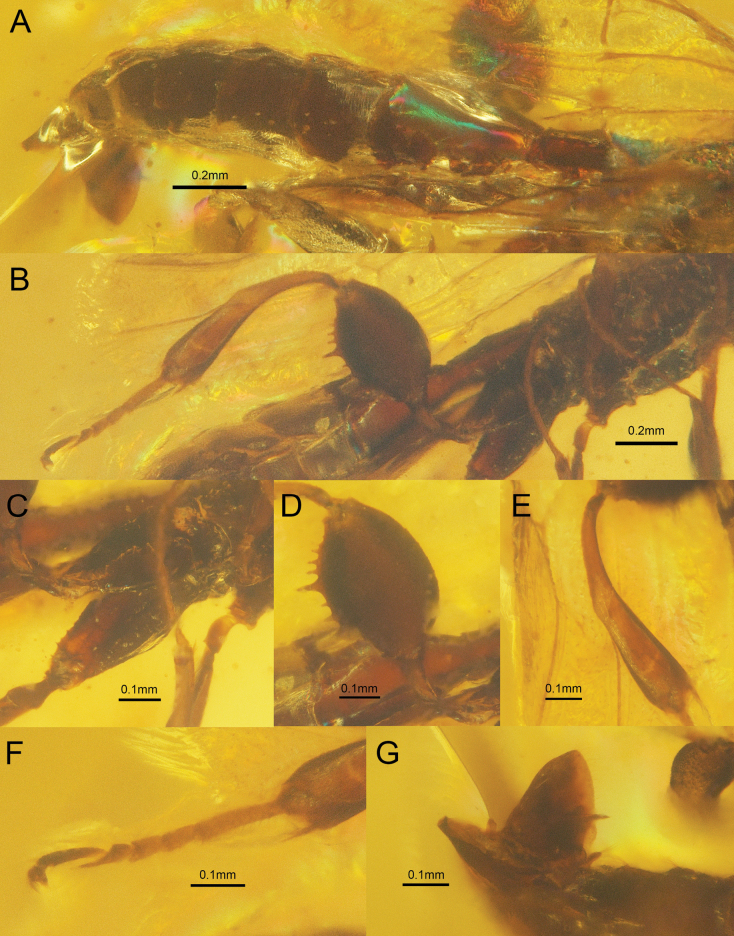
†*Tumidistephanusepimetheus* Ge & Tan, sp. nov. holotype ♂. A. Metasoma, lateral view; B. Hind leg, lateral view; C. Hind coxa, ventral-lateral view; D. Hind femur, lateral view; E. Hind tibia, lateral view; F. Hind tarsus, lateral view; G. Male genitalia.

***Mesosoma*.** Pronotum short and robust, lacking distinct pronotal fold; neck, medial and posterior region of pronotum aligned in profile. Pronotum and mesonotum densely foveate; mesopleuron laterally with oblique transverse rugosity and ventrally with large foveae.

**Figure 3.**
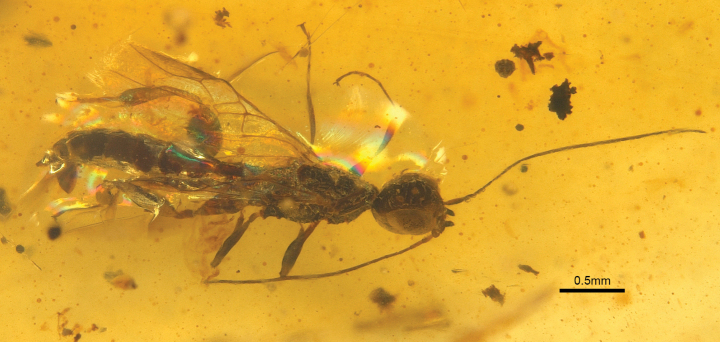
*Tumidistephanusepimetheus* Ge & Tan, sp. nov. holotype ♂.

***Wings*.** Forewing: vein 1-M 0.5 × as long as 1-Rs and slightly curved, 1.5 × as long as vein m-cu; vein 2-Rs 3.4 ×as long as vein r-rs; vein r-rs ends 0.3 ×length of pterostigma; vein A incomplete, reaching only reach at 1cu-a; vein Rs + M and 1Cu parallel; vein 2Rs+M almost absent, with no connection between veins 2-Rs and the distal segment of M. Hindwing with vein Cu-a and vein M+Cu lacking.

***Legs*.** Fore and mid legs with their femora and tibiae flattened and expanded. Metacoxa robust, spindle-shaped, ventrally with two rows of tubercles; metafemur coriaceous, extremely swollen medially, nearly oval in profile. Metafemur with two large and five medium-sized teeth (two intercalated between the large teeth, three discal to the apical large teeth); metatibia 1.2× as long as metafemur, with basal narrow portion 0.9× as long as apical broadened part; metatarsus with five tarsomeres; basitarsus 4.3× as long as wide.

***Metasoma*.** Metasoma with eight segments. First tergum and sternum not fused laterally, Tergite I rather robust, about 0.9× as long as tergite II.

**Female.** Unknown.

## ﻿Discussion

Ge et al. (2023) established the extinct subfamily †Lagenostephaninae, to accommodate a distinct lineage of Cretaceous Stephanidae, hypothesizing ecological specialization (e.g., potential parasitism of bark beetles) based on reduced body size and ovipositor length. The new species described herein, †*Tumidistephanusepimetheus* Ge & Tan, sp. nov., constitutes the fourth species assigned to †Lagenostephaninae and only the second male specimen known from the subfamily. Remarkably, †*T.epimetheus* exhibits a unique wing venation: veins Rs+M and 1Cu are strictly parallel—a trait not previously documented in other †Lagenostephaninae species. In contrast, the only other male specimen known from the subfamily, belonging to the species †*Neurastephanusneovenatus* Aguiar & Janzen, shows subparallel Rs+M and 1Cu, suggesting an intermediate condition. These differences in venation may indicate sexual dimorphism, a phenomenon not yet well documented within Stephanidae. We emphasize the necessity of incorporating male-based characters into future phylogenetic analyses to better resolve evolutionary relationships within this early-diverging hymenopteran lineage.

## Supplementary Material

XML Treatment for Tumidistephanus

XML Treatment for Tumidistephanus epimetheus

## ﻿Additional information

### Use of AI

No use of AI was reported.

### Author ORCIDs

Si-Xun Ge https://orcid.org/0000-0003-3769-1530

Li-Li Ren https://orcid.org/0000-0003-0333-0681

### Data availability

All of the data that support the findings of this study are available in the main text.
